# Lymphoepithelioma-like carcinoma of the stomach: a case report and review of the literature

**DOI:** 10.1186/1746-1596-8-184

**Published:** 2013-11-04

**Authors:** Zeid Bittar, Falko Fend, Leticia Quintanilla-Martinez

**Affiliations:** 1 Institute of Pathology, University Hospital Tuebingen, Eberhard-Karls-University, Liebermeisterstrasse 8, Tuebingen 72076, Germany

**Keywords:** Lymphoepithelioma-like carcinoma, EBV, Microsatellite instability, Prognosis

## Abstract

A lymphoepithelioma-like carcinoma (LLC), characterized by a carcinoma with stromal heavy lymphocyte infiltration, is one of the histological patterns observed in patients with Epstein-Barr virus (EBV)-associated gastric carcinoma. Although this entity is hard to be recognized in the biopsy specimens, pathologists and clinicians should acknowledge this subset of gastric cancer because it generally has a better prognosis than other forms of EBV-associated gastric carcinomas and conventional gastric carcinomas. This might be due to the fact that the patient's inflammatory response may prevent the spread of tumor through the gastric wall and to the lymph nodes or remote organs.

We report a case of EBV-positive, microsatellite stable LLC as a rare morphologic variant of gastric carcinoma.

**Virtual slides:**

The virtual slides for this article can be found here: http://www.diagnosticpathology.diagnomx.eu/vs/1360498724104351.

## Background

Lymphoepithelioma-like carcinoma (LLC) of the stomach is a rare and peculiar type of gastric carcinoma that was first described by Watanabe et al in 1976 as gastric carcinoma with a lymphoid stroma [1,2]. It constitutes about 4% of all gastric carcinomas [3,4]. Two subsets of gastric cancer, Epstein-Barr virus (EBV)-positive and microsatellite instability (MSI)-high cancers, have been associated with a lymphocyte-rich phenotype [5]. More than 80% of lymphoepithelioma-like gastric carcinomas have been found to be associated with EBV infection and express only several EBV-latent genes (Latency I program) [6] as opposed to 6% and 7% of diffuse and intestinal-type adenocarcinomas respectively [7]. The prevalence of MSI-high in gastric carcinomas ranges from 7% to 39% with geographic variability [5].

In this report, we highlight this rare variant of gastric cancer and discuss its association with EBV and the role of mismatch repair proteins in gastric LLC.

### Case presentation

This is a 68-year-old gentleman with past medical history of hypertension, hypothyroidism, depression, and panic attacks, presented with epigastric pain. No nausea, vomiting, hematemesis, or weight loss reported. He underwent endoscopic biopsy that showed a poorly differentiated adenocarcinoma. He was referred to a tertiary center for further evaluation. His physical examination was unremarkable. Laboratories were normal. Upper endoscopy revealed an ulcerated polypoid tumor involving the greater curvature. The endoscopic ultrasound (EUS) showed thickening of the gastric wall with destruction of the layered structure without infiltration or penetration of the serosa.

**Figure 1:**
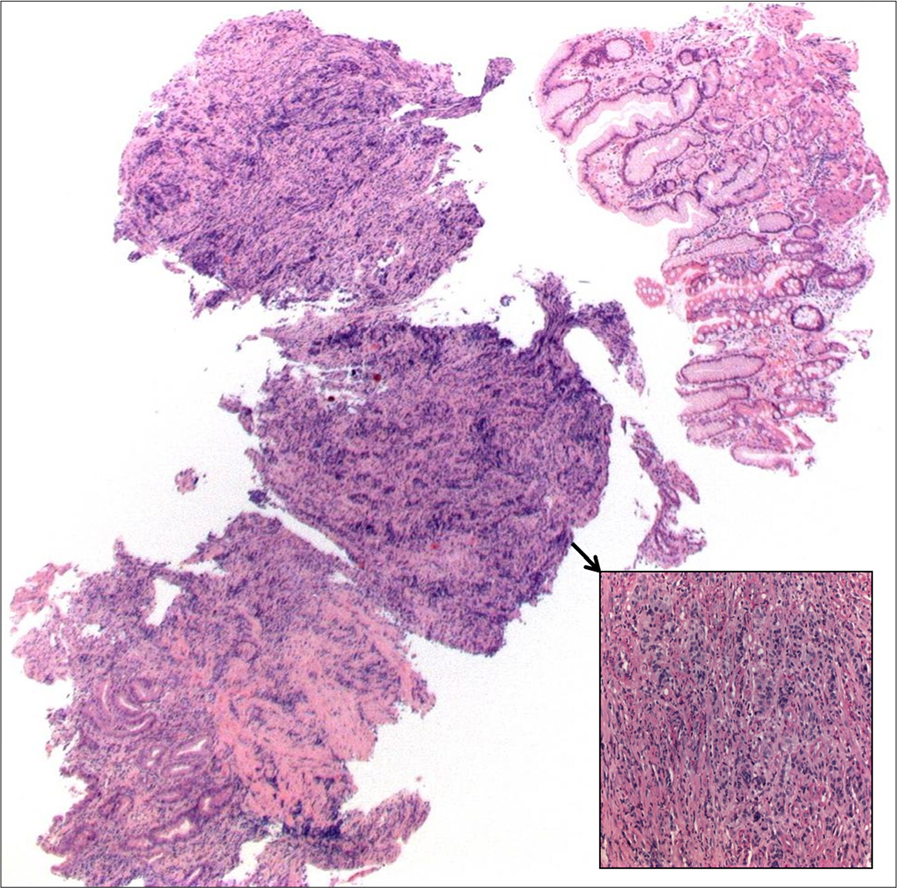
**Lymphoepithelioma-like gastric carcinoma (LLC) on biopsy specimens.** It is difficult to recognize the LLC in the biopsy specimens. In our case an outside diagnosis of poorly differentiated adenocarcinoma of the stomach was made, the diagnosis was also missed on the repeated biopsy in our institute too (HE Stain, Magnification 2.5X, insertion: PAS stain Magnification 20X).

The repeated biopsy revealed a carcinoma with solid proliferation and associated moderate chronic gastritis without evidence of Helicobacter pylori. The histological type of the tumor could not be identified (Figure 1). The dense stromal lymphocyte infiltrate thought to be superimposed probably secondary to the ulceration. The computed tomography (CT) was negative for ascites, lymph nodes enlargement, peritoneal carcinomatosis, or liver metastases. On the basis of these findings, and considering the patient’s age and general condition a gastrectomy was performed with lymph nodes dissection and a Roux-en-Y reconstruction.

The gross examination of the gastrectomy specimen revealed a patelliform tumor measuring 3.2 cm in diameter with depression and central ulcer. The cut section showed that the tumor invades the muscular layer of the gastric wall (the muscularis propria). Five centimeter apart from the tumor a 0.7 cm polypoid lesion was observed.

#### Histological, immunohistochemical and in situ hybridization findings

**Figure 2:**
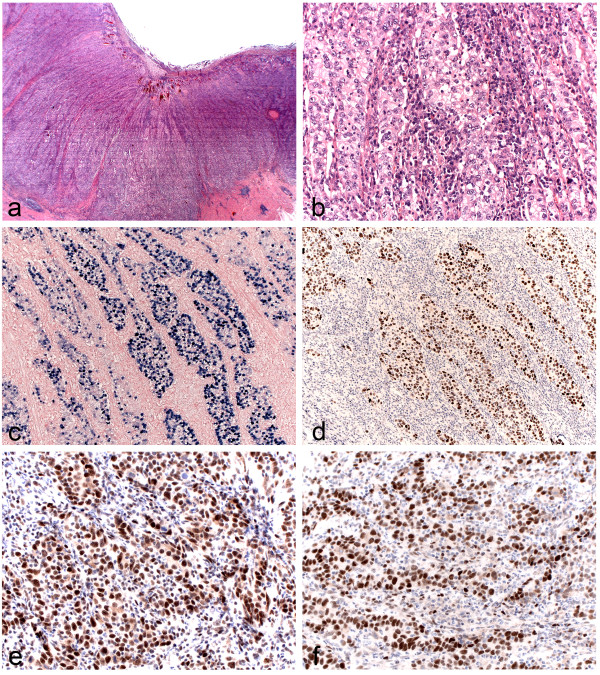
**LLC in association with EBV. (a.)** Loupe view of LLC. Note the well-circumscribed and expansive growth pattern with pushing borders (HE Stain, Magnification 2.5X). **(b.)** Microscopic view of LLC (HE Stain, Magnification 40X). **(c.)** EBER in situ hybridization in LLC (EBV-ISH, Magnification 40X). **(d.)** P53 positivity in the tumor cells (Magnification 40X). **(e.)** Retention of expression of the mismatch repair protein MLH1 and MSH6 **(f.)** (Magnification 40X).

Microscopically, the tumor was composed of sheets and nests of round to polygonal cells with poorly defined cell borders within a dense lymphocytic background. Cytologically, the tumor cells were large and pleomorphic with abundant eosinophilic cytoplasm, large vesicular nuclei and prominent nucleoli (Figure 2a and 2b). No vascular or lymphatic invasion was observed. No epithelioid cell granulomas or Mott cells identified. None of the 27 dissected lymph nodes showed metastasis. The accompanying small polypoid lesion at the greatest curvature was a hyperplastic polyp without adenomatous changes.

Immunohistochemical staining for pan-cytokeratin (CAM5.2 and AE1/AE3) was positive. The tumor was negative for CK7, CK20, synaptophysin, CDX2 and CD45.

The in situ hybridization for EBV-encoded small RNAs (EBER-1 and 2) showed an intensive nuclear hybridization signal corresponding to the carcinoma cells (Figure 2c), whereas the tumor was negative for both LMP1 and EBNA2. No EBV hybridization signal evident in the adjacent non-neoplastic gastric mucosa, in the lymphocytes around the tumor cell or in the dissected lymph nodes.

P53 showed strong and diffuse positivity in the tumor cells (Figure 2d). Regarding of the mismatch repair proteins there was retention of expression of all four of the proteins (MLH-1, PMS-2, MSH-2 and MSH-6) (Figure 2e and 2f).

Finally, EBV-associated LLC of the stomach was diagnosed and staged as (T2, N0, M0) according to the WHO classification of tumors 2010.

The clinical course was complicated on the 5^th^ postoperative day with insufficiency of the duodenal stump which necessitated revision, and at nine months with perforation of the left colonic flexure most probably of ischemic genesis which was managed by laparotomy and subtotal colectomy with side/side ileodescendostomy. Fifteen months after the operation the patient remains well with no evidence of recurrent disease.

## Discussion

LLCs are defined as tumors which possess histologic similarity to nasopharyngeal carcinoma [8]. LLC of the stomach is a rare type of gastric carcinoma that was first described by Watanabe et al as gastric carcinoma with a lymphoid stroma [1,2]. The stroma consists of CD8- or CD4-positive T lymphocytes, and CD68-positive macrophages, in a ratio of 2:1:1 and EBV infection is observed only in a very limited number of these infiltrating lymphocytes [6]. The reports in the literature with the synonyms *undifferentiated carcinoma with lymphoid stroma, gastric Lymphoepithelioma-like carcinoma, or medullary carcinoma* all describe carcinomas with similar morphology [9,10]. EBV-associated gastric LLC demonstrates a male predominance, and like most gastric carcinomas, occur in elderly people [11]. Although some reports suggest that the carcinoma occurs in patients of relatively younger age, meta analyses did not confirm this observation [6].

LLC is also known as EBV-associated gastric carcinoma. An etiologic association with EBV is based on the uniform expression of EBV in all tumor cells while absent in normal epithelium, dysplastic lesions [12] and lymphoid cells. The mechanism by which EBV contributes to carcinogenesis in gastric mucosa is still unknown [13,14]. EBV-positive gastric LLC has been confirmed to be composed of a monoclonal proliferation of a single EBV-infected progenitor cell. This has strongly suggested that EBV infects the gastric mucosa before neoplastic transformation and is involved in the early stage of gastric carcinogenesis [15-17].

The mechanism by which infection of the gastric epithelial cells occurs is also poorly understood. The EBV receptor, CD21, is not expressed on gastric mucosa, so that the virus may enter the cells through an alternative receptor or direct interaction of gastric epithelial cells with EBV-infected lymphocytes/ oropharyngeal epithelial cells [18] i.e. fusion-mechanism between EBV-infected lymphocytes and epithelial cells. This has been also supported by the fact that co-cultivation of virus producing lymphocytes shows higher efficiency of infection (up to 800-fold) than cell-free infection, therefore, EBV-infected epithelial cells, probably in the neck zone of fundic glands, are likely to initiate clonal growth to develop EBV-associated gastric carcinoma. Atrophic gastritis might induce the infiltration of EBV-carrying lymphocytes to increase the chance of contact with epithelial cells, or the inflammation may produce a cytokine-rich milieu to support the clonal growth of EBV-infected epithelial cells [6].

EBV is not integrated into the host DNA, but maintains itself as an episomal circular form in the nuclei of infected cells without the production of viral particles. EBV replicates synchronously with the host chromosomes at cell division. The descendent carcinoma cells, therefore, take over EBV-DNA of initially infected cells even at the fully developed stage of carcinoma [6].

The role of EBV in oncogenesis differs according to the host cell type and the immune status of the host. EBV-associated LLC belongs to the Latency I neoplasms, in which latent gene products are restricted to EBV nuclear antigen I (EBNA1), EBV-encoded small RNA (EBER), latent membrane protein 2A (LMP2A), and BamHI-A rightward transcripts (BARTs).

Representative viral proteins, EBNA2 and LMP1, are not expressed in Latency I neoplasms [6]. The immunohistochemical (LMP1 and EBNA2 negativity) and in situ hybridization findings (strong nuclear hybridization signal in tumor cells) in our case correspond to the Latency I program.

The primary molecular abnormality in EBV associated LLC of the stomach is global and non-random CpG island methylation in the promoter region of many cancer-related genes (such as LOX, HRASLS, FLNC, HAND1, and THBD) [19].

Experimental studies have demonstrated that viral LMP2A is responsible for the promotion of DNA methylation. LMP2A up-regulates cellular DNMT1 through the phosphorylation of STAT3, resulting in the repression of tumor suppressor genes, such as PTEN, through promoter methylation (immunohistochemistry confirmed the concurrent expression of pSTAT3 and DNMT1 in neoplastic cells of LLC gastric carcinoma in vivo [20]).

In regards to the CpG-island methylation phenotype (CIMP), gastric carcinoma has been classified into three subgroups: CIMP none, intermediate, and high (CIMP-N, -I, and -H) by the numbers of methylated loci: 0, 1-3, and 4 or more, respectively [21]. Nearly all cases of EBV associated LLC exhibited the CIMP-H phenotype and concurrently showed frequent CpG-island methylation in other cancer-related genes.

CpG-Methylation is not random in EBV-associated LLC. It showed significantly higher frequencies of methylation of cancer-related genes (p14ARF, p15, p16INK4A, p73, TIMP3, E-cadherin, DAPK, and GSTP1) than EBV negative/ CIMP-H LLC, except for the methylation of hMLH1, and MGMT.

The methylation of hMLH1 promoter downregulates its expression, causing a microsatellite instability (MSI)-high phenotype, and is the main inactivation mechanism of the *hMLH1* gene responsible for MSI-H in gastric carcinoma [22]. Several reports have reported that EBV infection and MSI-H are mutually exclusive in gastric carcinoma cases [5,23,24].

The prevalence of MSI-high in gastric carcinomas ranges from 7- 39% whilst a geographic variability has been found responsible for such a wide range [5].

Of the 3 cases that fulfilled the criteria for gastric LLC in the study by Leung et al [25], 2 were EBV positive but MSI negative, while the remaining case was EBV negative with a high level of MSI.

Ojima et al [26] examined the immunoexpression of p53 protein in EBV-positive and EBV-negative gastric cancers. Overexpression of p53 protein was demonstrated in only 8.4% of EBV-positive gastric cancers compared with 34.4% of EBV-negative cases. A study by Leung et al [27] found different results as all 18 EBV-associated gastric cancers were p53 positive. In the case under discussion the p53 protein was diffusely and strongly overexpressed in the tumor cells, suggestive of P53 mutations.

The predominant locations of EBV-associated gastric LLC are the cardia and middle portion of the stomach, while MSI-high associated gastric carcinomas are more common in the antrum [28]. The tumor in this case locates in the distal third of the corpus at the greater curvature.

LLC shows a lower rate of lymph node involvement, especially during its early stage within the submucosa. The incidence of multiple carcinomas also appears to be higher in EBV-associated gastric carcinoma than in EBV-negative carcinomas with EBV positivity reported to be higher in synchronous multiple carcinomas than in consecutive single carcinomas [6]. The tumor in our case was single and the lymph node involvement was not identified.

LLC of the stomach is known to have a favorable prognosis despite the fact that they are poorly differentiated carcinomas [12,29]. Watanabe et al. suggested that the lymphocytic infiltration in LLC was a host defense reaction against the cancer cells and that a more extensive lymphocytic infiltration was indicative of better prognosis [1]. It is unclear whether the survival advantage is related to the lymphoid infiltrate, or whether the EBV or MSI status itself serves as an independent prognosticator [5]. However, by analyzing a large series of patients, Beghelli et al concluded that the MSI phenotype significantly correlates with survival only in stage II neoplasms [30].

## Conclusion

We describe a case of EBV-positive, microsatellite stable LLC as a rare morphologic variant of gastric carcinoma, which has special clinical and histological features that distinguish it from other gastric adenocarcinomas. Although EBV is considered one of the causative factors in the development of this type of carcinomas, MSI is another one.

The diagnosis of lymphoepithelioma-like gastric carcinomas should mainly rest on the characteristic morphology and demonstration of EBV using different techniques. The MSI status (as detected by immunohistochemistry or PCR) is the other mainstay in the diagnosis with an important prognostic value.

LLC is difficult to recognize in the biopsy specimens as the stromal lymphocyte infiltrate always thought to be due to the ulceration. In spite, pathologists and clinicians should acknowledge this subset of gastric cancer because it generally has a better prognosis than other forms of EBV-associated gastric carcinomas and conventional gastric carcinomas.

## Consent

Written informed consent was obtained from the patient for publication of this case report and accompanying images. A copy of the written consent is available for review by the Editor-in-Chief of this journal.

## Abbreviations

LLC: Lymphoepithelioma-like carcinoma; EBV: Epstein-barr virus; MSI: Microsatellite instability; EBERs: EBV-encoded small RNAs; LMP: Latent membrane protein; EBNA: EBV nuclear antigen; BARTs: BamHI-A rightward transcripts.

## Competing interests

The authors declare that they have no competing interests.

## Authors’ contributions

ZB participated in conception of the idea, review of the literature, and writing of the manuscript. Q-ML participated in interpretation of the specimens. FF revised the manuscript critically for important intellectual content, and gave final approval of the version to be published. All authors read and approved the final manuscript.
